# A prognostic model for stratification of stage IB/IIA esophageal squamous cell carcinoma: a retrospective study

**DOI:** 10.1186/s12876-021-01636-5

**Published:** 2021-02-10

**Authors:** Lei-Lei Wu, Qi-Long Ma, Wei Huang, Xuan Liu, Li-Hong Qiu, Peng Lin, Hao Long, Lan-Jun Zhang, Guo-Wei Ma

**Affiliations:** 1 Sun Yat-Sen University Cancer Center, State Key Laboratory of Oncology in South China, Collaborative Innovation Center for Cancer Medicine, Guangzhou, 510060 People’s Republic of China; 2 Jinling Hospital, Nanjing, 210000 People’s Republic of China; 3 The Department of Thoracic Surgery, Sun Yat-Sen University Cancer Center, 651 Dongfengdong Road, Guangzhou, 510060 People’s Republic of China

**Keywords:** Esophageal squamous cell carcinoma, Stage IB/IIA, Lymph nodes, PD-L1, Prognosis

## Abstract

**Background:**

To explore the postoperative prognosis of esophageal squamous cell carcinoma (ESCC) patients with stage IB/IIA, using a prognostic score (PS).

**Methods:**

Stage IB/IIA ESCC patients who underwent esophagectomy from 1999 to 2010 were included. We retrospectively recruited 153 patients and extracted their medical records. Moreover, we analyzed the programmed death ligand-1 (PD-L1) expression of their paraffin tissue. The cohort were randomly divided into a training group (N = 123) and a validation group (N = 30). We selected overall survival (OS) as observed endpoint. Prognostic factors with a multivariable two-sided *P* < 0.05 met standard of covariate inclusion.

**Results:**

Univariable and multivariable analyses identified pTNM stage, the number of lymph nodes (NLNs) and PD-L1 expression as independent OS predictors. Primary prognostic score which comprised above three covariates adversely related with OS in two cohorts. PS discrimination of OS was comparable between the training and internal validation cohorts (C-index = 0.774 and 0.801, respectively). In addition, the PS system had an advantage over pTNM stage in the identification of high-risk patients (C-index = 0.774 vs. C-index = 0.570, *P* < 0.001). Based on PS cutoff, training and validation datasets generated low-risk and high-risk groups with different OS. Our three-factor PS predicted OS (low-risk subgroup vs. high-risk subgroup 60-month OS, 74% vs. 23% for training cohort and 83% vs. 45% for validation cohort).

**Conclusion:**

Our study suggested a PS for significant clinical stratification of IB/IIA ESCC to screen out subgroups with poor prognosis.

## Background

Esophageal carcinoma (EC) is a worldwide malignancy, ranking 9th and 6th in terms of incidence and mortality, respectively. About 509,000 EC cases die every year, and its major histological subtype is esophageal squamous cell carcinoma (ESCC) [1–4]. Over fifty per cent of new EC cases occur in China [5, 6], causing this country to present the highest mortality rate, with ESCC accounting for over 90% [6]. EC treatments include surgery, radiotherapy, chemotherapy, and immunotherapy, among which surgery remains the main treatment. Unfortunately, in China, the prognosis of surgical EC resection remains poor, with a 5-year survival rate of only 20–40% after esophagectomy [7]. Therefore, it’s important to screen out the patients with poor prognosis.

Both TNM stage and the number of lymph nodes (NLNs) dissected in surgery have been presented with clinical prognosis indicator in esophageal cancer [8]. In addition, there are still few predictors of EC development and prognosis [9–14]. Several previous studies reported that the expression of programmed death ligand-1(PD-L1) in lung cancer, breast cancer, and other tumors has a relation with the clinical significance of patients [15–22]. PD-L1 is a member of the B7-CD28 family, which is related to the tumor cell immune escape, playing an important role in induced T cell apoptosis [15, 21].

Here, we constructed a prognostic score (PS) system based on the TNM stage, NLNs, and expression of PD-L1, and they were independent prognostic indictors for OS. The current PS was able to divide the cohort into low- and high-risk subgroups, according to the survival outcome. This might provide clinically applicable information to give recommendations of follow-up management and monitoring.

## Methods

### Patients

**Fig. 1:**
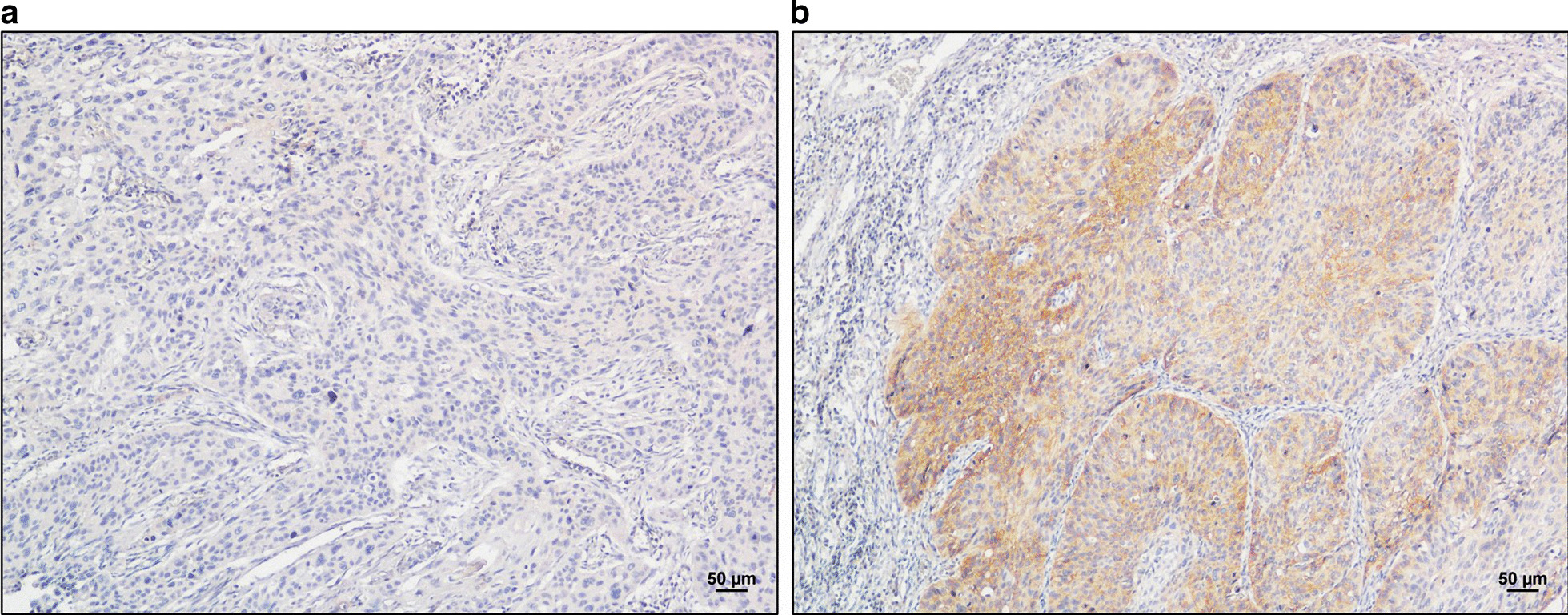
The diagram of this study

The Ethics Committee of Sun Yat-sen University Cancer Center (SYSUCC) approved the study’s protocol and exempted informed consent (approval number: YB2016-070). A total of 153 patients who underwent esophagectomy at the Department of Thoracic Surgery of SYSUCC between May 1999 and October 2010 were retrospectively enrolled in our study. Eligible cases had stage IB/IIA ESCC, pathologically confirmed according to the 8^th^ edition of the American Joint Committee on Cancer (AJCC) Staging Manual. The following exclusion criteria were applied: (1) patients who had received adjuvant and neoadjuvant cytotoxic chemotherapy or radiotherapy or immunotherapy regimens; (2) patients with a history of another malignant tumor; (3) patients with incomplete resection or margin residual tumor cells; (4) patients who died from postoperative complications or died within 1 month; (5) patients whose primary tumors were in the cervical esophagus or esophagogastric junction; and (6) patients with other pathological subtypes of EC besides ESCC. Included patients did not obviously present clinical evidence of inflammatory conditions. The pathological staging was translated into the 8^th^ edition of AJCC, using the patients’ records. The diagram of the study was presented with Fig. 1.

### Surgery

Surgeries were performed according to the following standard approaches of esophagectomy: McKeown (laparotomy, right thoracotomy, and neck incision), the Sweet (diaphragm incision and left thoracotomy), and the Ivor Lewis (right thoracotomy and laparotomy) procedures. Within the cohort, patients all performed thoracoabdominal dissection of lymph nodes. During surgery, the mean number of dissected lymph nodes (LNs) was 19.7.

### Follow-up

The median follow-up time was 97.9 months, with the last follow-up session being performed in May 2019. The patients were recommended to visit the outpatient department for follow-up every 3–6 months for the first 2 years, every 6–12 months for the next 3 years, and every year thereafter. The barium esophagography and neck–abdomen CT scans constituted the major follow-up examinations. Patients might undergo positron emission tomography-CT and/or endoscopy, as necessary.

### Immunohistochemical staining

Tumor and non-tumor paraffin tissues of all 153 patients were performed according to an Envision system of manufacturer’s instructions (Glostrup, Dako, Denmark). Polyclonal rabbit PD-L1 antibody (1:100; Cell Signaling Technology, Beverly, MA) and Ventana OmniMap anti-rabbit antibody were used as the primary and secondary antibodies, respectively. Staining intensity and extent were scored 0–3 and 0–4, respectively (0% for 0; 1–10% for 1 point; 11–25% for 2 points; 26–40% for three points; > 41% for 4 points). For each staining, the final quantitation was obtained by multiplying the two scores. The results of immunohistochemical staining were interpreted independently by two pathologists under double-blind conditions. They didn’t know any clinical and other pathological information. If the results were inconsistent, they would perform a joint discussion to decide the final result.

### Statistical analysis

Statistical analyses were performed using R version 3.5.2 (https://www.r-project.org/) and the SPSS Statistics 25.0 software (IBM SPSS, Inc., Chicago, IL, USA). The hazard ratio (HR) with 95% confidential interval (CI) were calculated by multivariate regression analysis. The cutoff value of PD-L1 expression and NLNs were determined using median, 3.0 and 16.0, respectively. According to above cutoff values, the PD-L1 expression ≤ 3 was regarded as low expression, and > 3 was defined as high expression. The associations between the PD-L1 expression, NLNs, and clinicopathological factors were assessed using the student’s t test, χ^2^ test and Fisher exact test. Standard error (SE) and standard deviation (SD)were used to evaluate the stability of continuous variables. Univariable analysis was performed to evaluate the influence of differentiation, pathological T stage, sex, pathological TNM stage, age, NLNs, smoking history, tumor length, drinking history, surgical approach, lymph node dissection of left recurrent laryngeal nerve, lymph node dissection of right recurrent laryngeal nerve, dissection of left gastric artery lymph node, dissection of subcarinal lymph node, and the level of PD-L1 expression on OS. A two-sided *P* < 0.05 was considered statistically significant. Multivariable analysis was used to select independent factors affecting OS. Variables were selected with univariable analysis of *P* < 0.05. In this study, we used one-way ANOVA test, linear regression and Pearson’s correlation analysis to explore the association between pathological TNM stage, NLNs and PD-L1 expression. The log-rank tests and Kaplan–Meier analysis were used to compare survival curves between groups. The model was developed and validated using a randomized method to extract trained and validated datasets. We used the function of “Random Sample of Cases” in SPSS, and set random sample size as 30. This randomized method made the ratio of training group to validation group 4:1.

Patients’ clinical characteristics and demographics were reported for the training group. The PS system for OS was constructed using three factors (NLNs, pTNM stage, and the expression of PD-L1), which was derived from the training dataset. The cohort was divided into a low-risk and a high-risk subgroup using median determine the PS cutoff value in the training cohort. A same cutoff value of risk score was defined to classify the patients in the internal validation cohort. C-index was used to estimate the discrimination of the multivariable survival prognostic model.

In the validation cohort, PS was applied to calculate the risk score, and classified patients into two subgroups, the low- and high-risk subgroups, basing on the same cutoff values defined in the training dataset.

## Results

The clinical variables of patients in the training and internal validation cohorts were shown in Table 1. Among the 153 patients, the 1-, 3- and 5-year OS rates were 84.0%, 71.0% and 46.0%, respectively. The patients’ age ranged from 37 to 81 years old (median, 60 years old). In the training group, the 1-, 3- and 5-year OS rates were 82.0%, 70.0% and 45.0%, respectively, and the median and mean survival times from surgery to the last censoring date were 91.9 and 82.0 months, respectively.

**Table 1:** The clinicopathologic characteristics of patients in the training and validation cohorts

Variable	All cohort	Training cohort		Validation cohort		*P value*
	N = 153 (%)	N = 123	%	N = 30	%	
Sex						0.248*
Male	115 (75.2)	90	73.2	25	83.3	
Female	38 (24.8)	33	26.8	5	16.7	
Age (year, mean ± SD)	59.7 ± 8.9	59.5 ± 9.0		60.5 ± 8.8		0.583**
Smoking status						0.157*
No	58 (37.9)	50	40.7	8	26.7	
Yes	95 (62.1)	73	59.3	22	73.3	
Drinking status						0.379*
No	107 (69.9)	88	71.5	19	63.3	
Yes	46 (30.1)	35	28.5	11	36.7	
Tumor length (cm, mean ± SD)	3.6 ± 1.5	3.7 ± 1.7		3.3 ± 1.0		0.227**
PD-L1 expression						0.780*
Low	80 (52.3)	65	52.8	15	50.0	
High	73 (47.7)	58	47.2	15	50.0	
Surgery approach						0.156*
Left thorax	108 (70.6)	90	73.2	18	60.0	
Right thorax	45 (29.4)	33	26.8	12	40.0	
Differentiation						0.013*
Well-moderate	71 (46.4)	51	41.5	20	66.7	
Poor	82 (53.6)	72	58.5	10	33.3	
pT stage						0.549*
T2	59 (38.6)	46	37.4	13	43.3	
T3	94 (61.4)	77	62.6	17	56.7	
NLNs (mean ± SD)	19.7 ± 14.1	19.3 ± 13.9		21.4 ± 14.9		0.470**
pTNM stage						0.102*
IB	52 (34.0)	38	30.9	14	46.7	
IIA	101 (66.0)	85	69.1	16	53.3	
LNs dissection of left recurrent laryngeal nerve						0.592*
No	47 (30.7)	39	31.7	8	26.7	
Yes	106 (69.3)	84	68.3	22	73.3	
LNs dissection of right recurrent laryngeal nerve						0.415*
No	45 (29.4)	38	30.9	7	23.3	
Yes	108 (70.6)	85	69.1	23	76.7	
Dissection of left gastric artery LNs						1.00***
No	9 (5.9)	7	5.7	2	6.7	
Yes	144 (94.1)	116	94.3	28	93.3	
Dissection of subcarinal LNs						0.69***
No	10 (6.5)	9	7.3	1	3.3	
Yes	143 (93.5)	114	92.7	29	96.7	

NLNs, the number of lymph nodes; LNs, lymph nodes

*P value was calculated by χ^2^ test; **P value was calculated by student’s *t* test; ***Fisher exact test

**Fig. 2:**
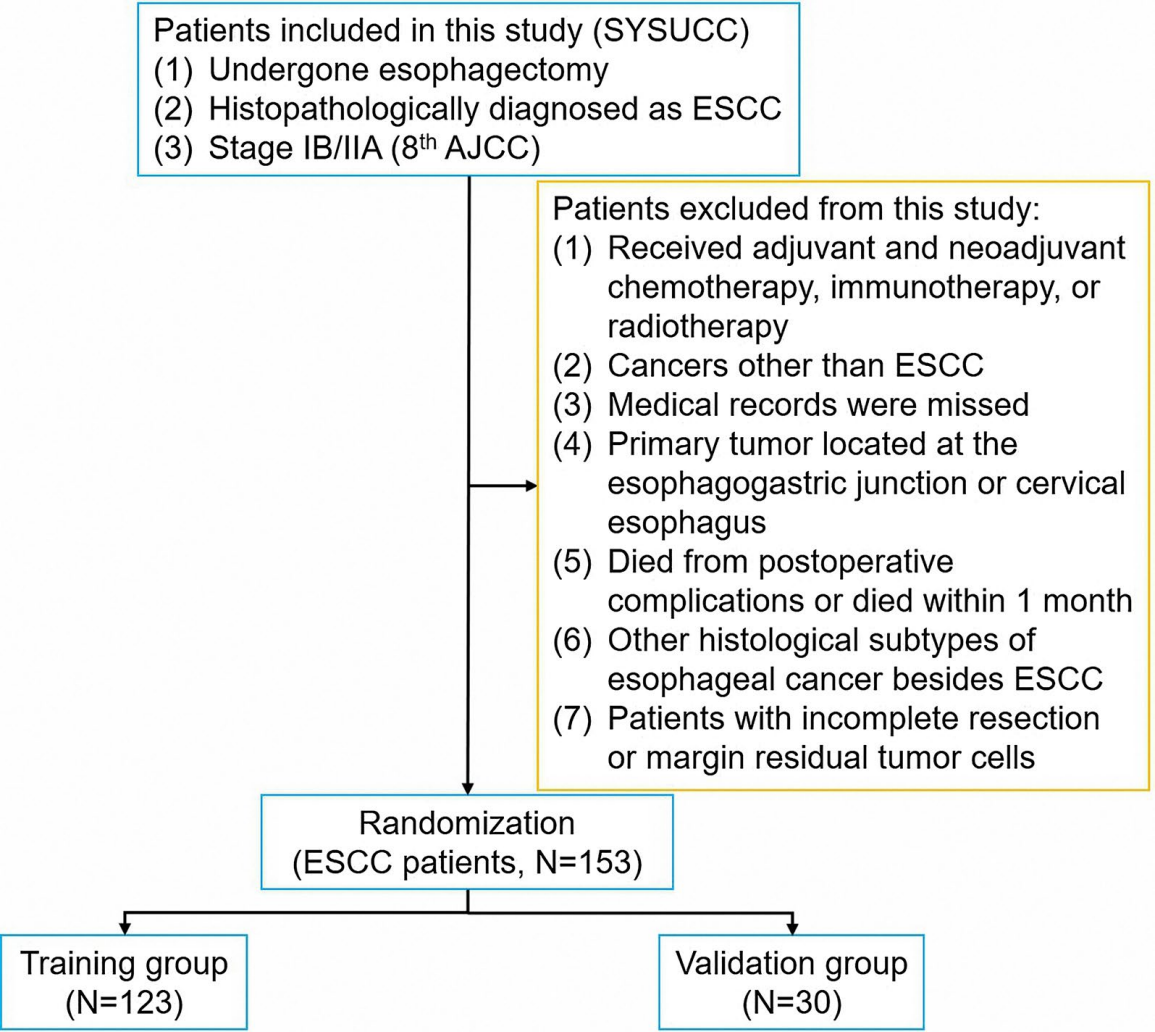
Immunohistochemical staining of PD-L1 in ESCC paraffin tissue with stage IB/IIA (**a** Low expression of PD-L1, staining intensity score was 1 point, staining extent score was 1 point, final score was 1 point; **b** High expression of PD-L1, staining intensity score was 3 points, staining extent score was 2 points, final score was 6 points)

**Fig. 3:**
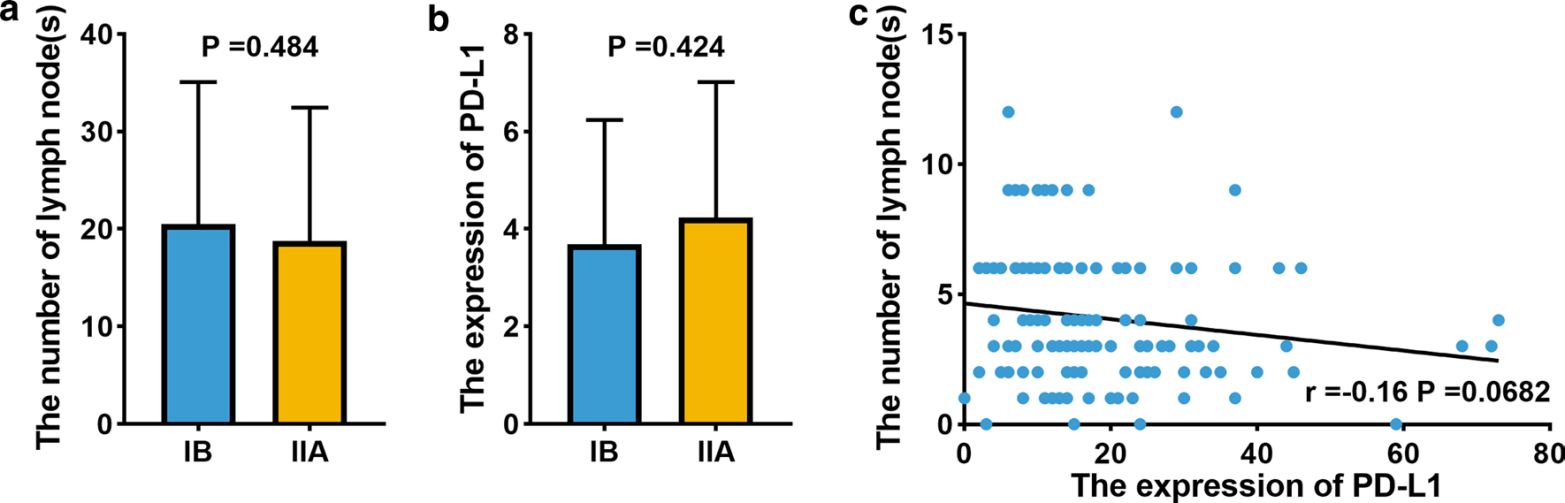
**a** Correlation between TNM stage and the number of lymph nodes (NLNs) (one-way ANOVA test); **b** Correlation between TNM stage and PD-L1 expression (one-way ANOVA test); C. Correlation between NLNs and PD-L1 expression (linear regression and Pearson’s correlation analysis)

Within the training cohort, a high level of PD-L1 expression was found in 58 of the 123 (47.2%) cases, and the expression of PD-L1 was shown as Fig. 2. The significance of PD-L1 and NLNs in ESCC was verified by correlating the status of PD-L1 and NLNs in 123 ESCC cases with widely recognized clinicopathological features (Table 2). Our results suggest that NLNs is correlated with surgical approach (Table 2). Univariable and multivariable analyses were performed to identify correlations between clinical characteristics and OS. As shown in Table 3, univariable and multivariable analyses identified the following clinical factors as significant OS prognostic indictors in patients with ESCC: NLNs (adjusted HR 0.963, 95%CI 0.938–0.989, *P* = 0.006), pTNM stage (adjusted HR 1.987, 95%CI 1.050–3.761, *P* = 0.035), and the expression of PD-L1 (adjusted HR 4.746, 95%CI 2.669–8.438, *P* < 0.001). The association of above three factors was shown in Fig. 3. We found that there was no statistically significant correlation among NLNs, pTNM stage, and the expression of PD-L1. In addition, our study showed that the level of PD-L1 expression, NLNs, and pTNM stage were significantly associated with OS in patients of ESCC.

**Table 2:** Correlation between PD-L1 expression, NLNs and clinicopathological characteristics in training cohort

Variable	PD-L1 expression		*P value*	NLNs		*P value*
	Low	High		≤ 16	> 16	
	N = 65 (%)	N = 58 (%)		N = 66 (%)	N = 57 (%)	
Sex			0.819*			0.773*
Male	47 (72.3)	43 (74.1)		49 (74.2)	41 (71.9)	
Female	18 (27.7)	15 (25.9)		17 (25.8)	16 (28.1)	
Age (years, mean ± SD)	59.5 ± 8.9	59.5 ± 9.1	0.980**	60.0 ± 7.9	59.0 ± 10.0	0.544**
Smoking status			0.832*			0.424*
No	27 (41.5)	23 (39.7)		29 (43.9)	21 (36.8)	
Yes	38 (58.5)	35 (60.3)		37 (56.1)	36 (63.2)	
Drinking status			0.549*			0.625*
No	48 (73.8)	40 (69.0)		46 (69.7)	42 (73.7)	
Yes	17 (26.2)	18 (31.0)		20 (30.3)	15 (26.3)	
Tumor length (cm, mean ± SD)	3.8 ± 1.6	3.6 ± 1.7	0.440**	3.6 ± 1.5	3.8 ± 1.8	0.584**
Surgical approach			0.525*			0.002*
Left thorax	46 (70.8)	44 (75.9)		56 (84.8)	34 (59.6)	
Right thorax	19 (29.2)	14 (24.1)		10 (15.2)	23 (40.4)	
NLNs			0.077*			–
≤ 16	30 (46.2)	36 (62.1)		–	–	
> 16	35 (53.8)	22 (37.9)		–	–	
Differentiation			0.701*			0.385*
Well-moderate	28 (43.1)	23 (29.7)		25 (37.9)	26 (45.6)	
Poor	37 (56.9)	35 (60.3)		41 (62.1)	31 (54.4)	
pT stage			0.796*			0.215*
T2	25 (38.5)	21 (36.2)		28 (42.4)	18 (31.6)	
T3	40 (61.5)	37 (63.8)		38 (57.6)	39 (68.4)	
pTNM stage			0.254*			0.811*
IB	23 (35.4)	15 (25.9)		21 (31.8)	17 (29.8)	
IIA	42 (64.6)	43 (74.1)		45 (68.2)	40 (70.2)	
PD-L1 expression			–			0.077*
Low	–	–		30 (45.5)	35 (61.4)	
High	–	–		36 (54.5)	22 (38.6)	
LNs dissection of left recurrent laryngeal nerve			0.813*			0.454*
No	20 (30.8)	19 (32.8)		19 (28.8)	20 (35.1)	
Yes	45 (69.2)	39 (67.2)		47 (71.2)	37 (64.9)	
LNs dissection of right recurrent laryngeal nerve			0.416*			0.586*
No	18 (27.7)	20 (34.5)		19 (28.8)	19 (33.3)	
Yes	47 (72.3)	38 (65.5)		47 (71.2)	38 (66.7)	
Dissection of left gastric artery LNs			0.706***			0.121***
No	3 (4.6)	4 (6.9)		6 (9.1)	1 (1.8)	
Yes	62 (95.4)	54 (93.1)		66 (90.9)	56 (98.2)	
Dissection of subcarinal LNs			0.498***			0.732***
No	6 (9.2)	3 (5.2)		4 (6.1)	5 (8.8)	
Yes	59 (90.8)	55 (94.8)		62 (93.9)	52 (91.2)	

NLNs, the number of lymph nodes

*P value was calculated by χ^2^ test; **P value was calculated by student’s *t* test; ***Fisher exact test

**Table 3:** Univariable and multivariable Cox proportional hazard regression analyses of the characteristics in training cohort

Variables	Univariable analysis			Multivariable analysis		
	*HR*	95% CI	*P* value	*HR*	95% CI	*P* value
Sex						
Male	1 (reference)					
Female	1.170	0.665–2.061	0.586			
Age (year, continuous)	1.024	0.995–1.053	0.107			
Smoking status						
No	1 (reference)					
Yes	1.305	0.764–2.231	0.330			
Drinking status						
No	1 (reference)					
Yes	1.627	0.949–2.790	0.077			
Differentiation						
Well-moderate	1 (reference)					
Poor	0.681	0.406–1.140	0.144			
pT stage						
T2	1 (reference)					
T3	1.677	0.952–2.954	0.073			
pTNM stage						
IB	1 (reference)			1 (reference)		
IIA	2.080	1.101–3.930	0.024	1.987	1.050–3.761	0.035
Tumor length (cm, continuous)	1.031	0.889–1.197	0.684			
Surgery approach						
Left	1 (reference)					
Right	0.624	0.330–1.179	0.146			
NLNs (continuous)	0.960	0.935–0.985	0.002	0.963	0.938–0.989	0.006
PD-L1 expression						
Low	1 (reference)			1 (reference)		
High	4.910	2.770–8.702	< 0.001	4.746	2.669–8.438	< 0.001
Dissection of left gastric artery LNs						
No	1 (reference)					
Yes	4.338	0.599–31.44	0.146			
Dissection of subcarinal LNs						
No	1 (reference)					
Yes	0.965	0.349–2.668	0.946			
LNs dissection of left recurrent laryngeal nerve						
No	1 (reference)					
Yes	1.362	0.756–2.452	0.304			
LNs dissection of right recurrent laryngeal nerve						
No	1 (reference)					
Yes	1.128	0.634–2.008	0.681			

Multivariable analysis’s method is Forward: LR

HR, hazard ratio; CI, confidential interval; NLNs, the number of lymph nodes; LNs, lymph nodes

**Fig. 4:**
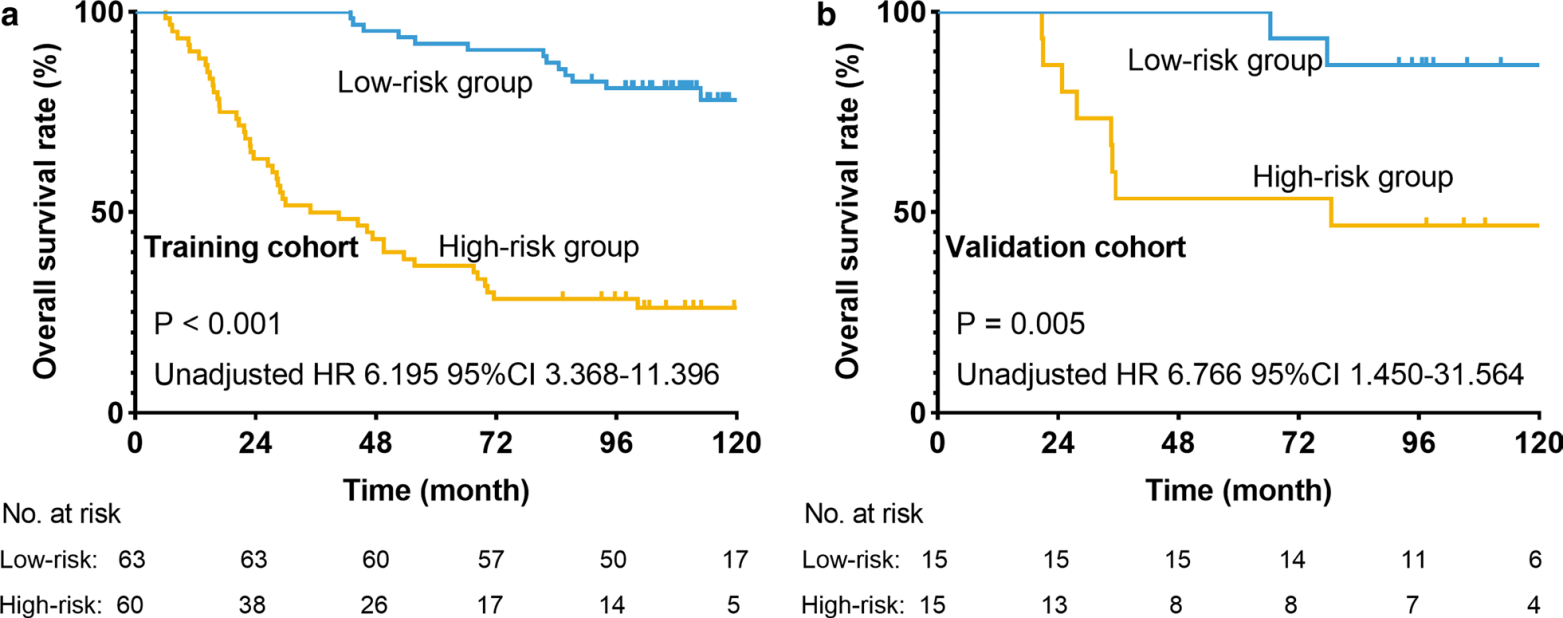
Overall survival curve for cohort of patients with stage IB/IIA ESCC according to the PS (**a** training cohort; **b** validation cohort)

Based on the results of the training cohort information analysis, we constructed the PS system and tested the covariates listed in Table 4 for their relation with OS. The PS system was based on weighting (derived by the β-coefficient of the respective log[HRs]) of the three significant covariates in the training group (Table 4), which generated C-index of 0.774 ± 0.029 for OS. In fact, in the training group, our PS included pTNM stage, NLNs and the expression of PD-L1 had a more exact predictive ability than pTNM stage for 5-year OS (PS: C-index = 0.774, TNM stage: C-index = 0.570, *P* < 0.0001). In other words, the PS system had an advantage over pTNM stage in the discrimination of high-risk patients. This model allowed us to define a low-risk subgroup presenting a significantly increased likelihood of survival (unadjusted HR 6.195, 95% CI, 3.368–11.396; *P* ˂ 0.001, Fig. 4a). The PS cutoff value was determined to distinguish between the high-risk and low-risk subgroups, using the median 107.0.

**Table 4:** Constructed prognostic score to predict overall survival in stage IB/IIA ESCC patients

Covarite	β [HR = exp (β)]	Score
NLNs	− 0.038	− 0.038 * continuous
PDL-1 expression	1.557	1.557 * (low expression = 0, high expression = 1)
pTNM stage	0.687	0.687 * (IB = 1, IIA = 2)
Total computed score and risk stratification		total score *100
Low risk		≤ 107.0
High risk		> 107.0

NLNs, the number of lymph nodes

In the validation group, the 1-, 3- and 5-year OS rates were 93.0%, 77.0% and 54.0%, respectively, and the median and mean survival times were 98.2 and 94.1 months, respectively. To validate the PS’s predictive accuracy for OS in IB/IIA ESCC, we examined the PS in the internal validation cohort: a cohort of 30 cases. The same PS cutoff value of 107.0 allowed us to stratify the patients within the validation cohort into either a low-risk subgroup with a significantly better OS or a high-risk subgroup (unadjusted HR 6.766, 95% CI, 1.450–31.564; *P* = 0.005, Fig. 4b). The PS in the internal validation dataset yielded C-index of 0.801 ± 0.061 for OS.

## Discussion

IB/IIA stage ESCC is the disease without metastases of lymph nodes, which is seen as early stage disease. Guidelines doesn’t recommend that these postoperative patients need to undergo the adjuvant treatment, such as chemotherapy and radiotherapy. However, the occurrence and development of ESCC is complex, and prognosis in a part of postoperative ESCC patients with stage IB/IIA is poor. The present study aimed to provide useful information to screen out the patients with poor prognosis. The patients’ clinical information and immunohistochemistry were analyzed, including the indicators shown in Table 1. Three meaningful indicators, NLNs, pTNM stage and PD-L1 expression levels, were selected through univariable and multivariable analyses of the training set. We constructed a prognostic model based on NLNs, pTNM stage and PD-L1 expression and successfully identified high- and low-risk populations within the training and validation cohorts. Our model has a significant effect on patients’ differentiation (Fig. 4), as the C-index predicting the OS rate reaches 0.801 and 0.774 in the internal validation and training sets, respectively. In fact, in the training group, our PS had a significant improvement than pTNM stage for predictive ability of 5-year OS (PS: C-index = 0.774, TNM stage: C-index = 0.570, *P* < 0.0001). Of note, the PS system had an advantage over pTNM stage in the discrimination of high-risk patients. According to our findings, patients with high risk score might require close attention from doctors and they would better be recommended to choose a shorter follow-up interval from what guidelines suggest [23]. In terms of clinical application, routine postoperative pathological records include tumor invasion, and NLNs. Evaluation of PD-L1 expression requires only immunohistochemical staining of postoperative paraffin tissues and the respective interpretation, independently performed by two pathologists. Therefore, NLNs, pTNM stage and PD-L1 expression levels can be clinically measured, thus facilitating the model’s wide range of applications.

We found that the differentiation of tumor had a difference between training and validation groups after random grouping (Table 1). However, the tumor differentiation was excluded using univariable and multivariable analyses, so the tumor differentiation had no effect in building our PS (Table 3). This was likely to be due to the low number of cases included in the study. Accordingly, we suggested that the degree of tumor differentiation had no effect on OS because of uneven distribution and small sample size. In addition, the C-index of validation cohort was higher than training cohort. Given the small sample size of validation group, we found that there was likely to be an impact of “overfitting” in the process of statistics. As the median of the whole data was used as the cutoff value of, which made the results of our study more objective, we used the median as the cutoff of PD-L1 expression and NLNs.

There are some limitations in the present study. First, it is a single-institution study with a small sample size. It is therefore necessary to expand the results by performing multicenter studies with larger cohorts. Since ESCC is the main pathological type in China, the present study did not include patients with adenocarcinoma. Second, given “overfitting” might affect the results of validation group and the median was regarded as cutoff value of PD-L1, NLNs and PS, more cases are needed to further explore more appropriate statistic methods and more exact results and cutoff value. Third, since only patients with stage IB/IIA ESCC were enrolled, this model cannot predict or evaluate the prognosis of patients with lymph node metastasis and can only be applied to IB/IIA ESCC patients.

## Conclusions

In conclusion, the PD-L1 expression, pTNM stage and NLNs were independent prognostic indictors for ESCC in stage IB/IIA. In addition, we present a validated PS for robust clinical stratification of IB/IIA ESCC to screen subgroups with poor prognosis. The PS had a significant improvement than pTNM stage for predictive ability of 5-year OS. Our PS may provide useful information to screen out the patients of poor prognosis. However, more studies are needed to explore the effect of PS on prognosis of ESCC patients in stage IB/IIA.

## Data Availability

Researchers interested in this study may contact the authors to obtain the clinical data of all 153 patients. We have uploaded the data of this study in the Research Data Deposit of SYUCC, and its number was RDDA2019001167 (http://www.researchdata.org.cn/).

## Abbreviations

ESCC: Esophageal squamous cell cancer
PS: Prognostic score
NLNs: The number of lymph nodes
OS: Overall survival
SYSUCC: Sun Yat-sen University Cancer Center
HR: Hazard ratio
CI: Confidential interval
EC: Esophageal cancer
PD-L1: Programmed death ligand-1
AJCC: American Joint Committee on Cancer
SD: Standard deviation
SE: Standard error
LNs: Lymph nodes

## References

[1] Bray F, Ferlay J, Soerjomataram I (2018). Global cancer statistics 2018:GLOBOCAN estimates of incidence and mortality worldwide for 36 cancers in 185 countries. CA Cancer J Clin.

[2] Arnold M, Soerjomataram I, Ferlay J, Forman D (2015). Global incidence of oesophageal cancer by histological subtype in 2012. Gut.

[3] Arnold M, Laversanne M, Brown LM (2017). Predicting the future burden of esophageal cancer by histological subtype: international trends in incidence up to 2030. Am J Gastroenterol.

[4] Edgren G, Adami HO, Weiderpass E, Nyren O (2013). A global assessment of the oesophageal adenocarcinoma epidemic. Gut.

[5] Abnet CC, Arnold M, Wei WQ (2018). Epidemiology of esophageal squamous cell carcinoma. Gastroenterology.

[6] Liang H, Fan JH, Qiao YL (2017). Epidemiology, etiology, and prevention of esophageal squamous cell carcinoma in China. Cancer Biol Med.

[7] Du L, Wei W (2018). Progress research in survival analysis of esophageal squamous cell carcinoma (ESCC) in Chinese population. J Pract Oncol.

[8] Zhou L, Zhao Y, Zheng Y (2020). The prognostic value of the number of negative lymph nodes combined with positive lymph nodes in esophageal cancer patients: a propensity-matched analysis. Ann Surg Oncol.

[9] Deng J, Weng X, Ye J (2019). Identification of the germline mutation profile in esophageal squamous cell carcinoma by whole exome sequencing. Front Genet.

[10] Wu LL, Liu X, Huang W (2020). Preoperative squamous cell carcinoma antigen and albumin serum levels predict the survival of patients with stage T1–3N0M0 esophageal squamous cell carcinoma: a retrospective observational study. J Cardiothorac Surg.

[11] Kiyozumi Y, Baba Y, Okadome K (2019). IDO1 expression is associated with immune tolerance and poor prognosis in patients with surgically resected esophageal cancer. Ann Surg.

[12] Huang W, Wu L, Liu X (2019). Preoperative serum C-reactive proteinlevels and postoperative survival in patientswith esophageal squamous cell carcinoma: a propensity score matching analysis. J Cardiothorac Surg.

[13] Tseng RC, Chang JM, Chen JH (2015). Deregulation of SLIT2-mediated Cdc42 activity is associated with esophageal cancer metastasis and poor prognosis. J Thorac Oncol.

[14] Yamamura K, Baba Y, Nakagawa S (2016). Human microbiome fusobacterium nucleatum in esophageal cancer tissue is associated with prognosis. Clin Cancer Res.

[15] Iwai Y, Ishida M, Tanaka Y (2002). Involvement of PD-L1 on tumor cells in the escape from host immune system and tumor immunotherapy by PD-L1 blockade. PNAS.

[16] Choueiri TK, Fay AP, Gray KP (2014). PD-L1 expression in nonclear-cell renal cell carcinoma. Ann Oncol.

[17] D'Angelo SP, Shoushtari AN, Agaram NP (2015). Prevalence of tumor-infiltrating lymphocytes and PD-L1 expression in the soft tissue sarcoma microenvironment. Hum Pathol.

[18] Gao Y, Li S, Xu D (2017). Prognostic value of programmed death-1, programmed death-ligand 1, programmed death-ligand 2 expression, and CD8(+) T cell density in primary tumors and metastatic lymph nodes from patients with stage T1–4N+M0 gastric adenocarcinoma. Chin J Cancer.

[19] Madore J, Vilain RE, Menzies AM (2015). PD-L1 expression in melanoma shows marked heterogeneity within and between patients: implications for anti-PD-1/PD-L1 clinical trials. Pigment Cell Melanoma Res.

[20] Muenst S, Schaerli AR, Gao F (2014). Expression of programmed death ligand 1 (PD-L1) is associated with poor prognosis in human breast cancer. Breast Cancer Res Treat.

[21] Topalian SL, Hodi FS, Brahmer JR (2012). Safety, activity, and immune correlates of anti-PD-1 antibody in cancer. N Engl J Med.

[22] Zhang J, Fang W, Qin T (2015). Co-expression of PD-1 and PD-L1 predicts poor outcome in nasopharyngeal carcinoma. Med Oncol.

[23] National Comprehensive Cancer Network. Esophageal and Esophagogastric Junction Cancers (Version 4.2020). https://www.nccn.org/professionals/physician_gls/pdf/esophageal.pdf Accessed 14 Aug 2020.

